# Increasing the evidence base in journalology: creating an international best practice journal research network

**DOI:** 10.1186/s12916-016-0707-2

**Published:** 2016-10-10

**Authors:** David Moher, Philippe Ravaud

**Affiliations:** 1 Centre for Journalology, Clinical Epidemiology Program, Ottawa Hospital Research Institute, The Ottawa Hospital, General Campus, 501 Smyth Rd, Room L1288, Ottawa, ON K1H 8 L6 Canada; 2 School of Epidemiology, Public Health and Preventive Medicine, University of Ottawa, Ottawa, Canada; 3 METHODS Team, Epidemiology and Statistics Sorbonne Paris Cité, Research Center INSERM UMR 1153, Paris Descartes University, Paris, France

**Keywords:** Journalology, Editors, Publishers, Funders, Research, Evidence, Best practice, Quality of reporting

## Abstract

Biomedical journals continue to be the single most important conduit for disseminating biomedical knowledge. Unlike clinical medicine, where evidence is considered fundamental to practice, journals still operate largely in a ‘black box’ mode without sufficient evidence to drive their practice. We believe there is an immediate need to substantially increase the amount and quality of research by journals to ensure their practice is as evidence based as possible. To achieve this goal, we are proposing the development of an international ‘best practice journal research network’. We invite journals and others to join the network. Such a network is likely to improve the quality of journals. It is also likely to address many unanswered questions in publication science, including peer review, which can provide robust and generalizable answers.

## Background

Biomedical journals remain the central conduit through which biomedical knowledge is disseminated; this is likely to continue for the foreseeable future regardless of the publishing models used for dissemination. However, even a cursory scan of what journals publish raises concern – the quality of reporting of peer-reviewed and editor-approved clinical and preclinical research is appalling [1–5]. If reports of clinical research are unusable, this substantially reduces the confidence clinicians have in using best evidence to inform best practice, therefore affecting patient care directly.

The reasons behind the poor completeness of reporting of biomedical research are complex and involve various players - publishers, journals, and scientific editors are partially responsible. For example, editors do not explicitly recommend the use of reporting guidelines as part of the review process [6] despite emerging evidence indicating that their use is associated with more complete reporting [7, 8]. Further, there appears to be a knowledge gap for scientific editors and peer reviewers [9]; more than a third of manuscripts submitted to 46 journals for publication consideration were inappropriately classified by the editorial offices as randomized trials during a study [10]. Additionally, there is still no agreement regarding the effectiveness of peer review. Unlike clinical medicine, where evidence is considered fundamental to practice, journals still operate largely in a ‘black box’ mode without sufficient evidence to drive their practice.

Innovative editors have not stood still. In 1989, championed by Drummond Rennie and *JAMA*, the first International Congress on Peer Review and Biomedical Publication was held, its remit being to investigate and better understand the scientific process of publication. The congress, held every 4 years, and jointly coordinated with the *BMJ*, is an important oracle for sharing journalology [11] research. The eighth congress will be held in Chicago in 2017 [12]. Likewise, in 1994, Richard Smith, editor-in-chief of BMJ established Locknet, its remit being the focus on research issues related to the peer review process [13]. Locknet is not currently active (Richard Smith, personal communication), although the *BMJ* and *JAMA* both have a long tradition of collaborative journalology research.

The evidence base underlying journalology is too small and consists largely of descriptive studies and few observational and quasi-experimental studies [14]. An underwhelming number of randomized trials [14] have addressed ‘what works’ questions in journalology and in a related field of investigation, research on research (i.e., meta-research) [15]. Therefore, we are interested in finding ways to substantially increase the amount of research conducted by journals in journalology and meta-research. We believe there are models in clinical medicine that could be successfully adapted for journals to develop and sustain an international biomedical journal research network (http://or.org/pdf/NIH_Roadmap-ClinicalResearch.pdf). Similarly, the Army of Women (https://www.armyofwomen.org/) has proven to be an effective model to increase participation in breast cancer trials. This might be a useful model to consider in order to ensure the participation of sufficient journals in journalology research.

It is likely that many journal editors have limited experience in participating in research studies and may feel they lack the expertise to conduct and participate in such studies. Therefore, we think that any journal wanting to join the network should ‘buddy’ with an established research team involved in this domain. Journals are best placed to consider how such an arrangement might work optimally and the arrangements might differ across journals; one option to consider is appointing a member of the clinical research team as a member of a journal’s scientific editorial team (http://eyes.cochrane.org/partnerships-eyes-and-vision-journals).

Journals might be interested in joining the network, and conducting and participating in research, for a variety of reasons – it is likely the best way of improving the quality of their journal (clinical centres participating in research provide better care and participants have better outcomes) [16]; it would enhance journal personnel expertise and experience in research, including clinical trials, which in turn might also help them review similar submissions to their journals; and it would help to more reliably answer relevant questions about journalology.

A journal research network can play an important role in enhancing the science of journalology and meta-research. Many researchers have addressed questions around whether peer review is effective. To date, there is no clear definitive answer. Too few studies have been conducted and, of those published, there is little agreement about optimal designs [17] and best primary outcome(s). Our community might benefit from experiences in clinical medicine by developing core outcome sets across a range of journalology topics, including peer review, and participating in the COMET initiative [18]. Similarly, we might benefit from involving members of the public and other advocacy groups in our research, following similar developments in clinical medicine.

We have discussed the idea of developing a network of journals for conducting research with a few colleagues in leadership positions at several journals (see Acknowledgements). These journals have welcomed this development and have agreed to help establish the initiative. While editors are crucial to help ensure the development and success of the network, other groups are also needed. Perhaps editors can promote the network to their publishers. Publishers bring an important cultural and policy authority to the table; they can be a powerful voice in enabling new perspectives such as data sharing. Similarly, they will be an important voice in taking new evidence generated from the network and implementing best practices in their journal(s). Funders of biomedical research are also important to include in the network. Their investment in research has often resulted in substantial waste in the form of unusable publications [19]. It is safe to assume their interests lie in increasing the research value of their investments, ensuring the public’s funding of research is being used optimally.

By joining the network, journals commit to engage in discussions about ways to improve the quality of published research. Since this initiative is new, it is difficult to be more precise as to how the network will evolve and mature over time. However, for the immediate future, the practical goals are to meet virtually, gauge interest in developing audit and feedback mechanisms to monitor the quality of journal publications, and provide opportunities for journals to participate in various research projects. The primary remit of the network is to substantially increase the amount of research, including clinical trials, addressing relevant questions in journalology. We invite journals to join the “Best Practice Journal Research Network” by completing a simple registration form (http://www.bpjrn.com/). An important outcome of this increased activity will be to provide more data and robust research to inform an evidence-based approach to running the scientific aspects of journals.

There is still a large number of unanswered questions that research can provide robust and generalizable answers to (Box 1). We know there are many other questions that need to be answered and we invite others to submit questions to the network website. This can provide a venue to refine the research question and proposed methodology, and to conduct and report such research optimally. Similarly, in clinical medicine, multi-centre research often provides greater generalizability of the results. While conducting research at a single journal is a good starting point, we believe such research is more generalizable when conducted across multiple journals. We think a network of journals working together will increase the number of multi-centre studies in journalology.

Recognizing the importance of access to data for biomedical research, editorial groups are now advocating for this in clinical medicine [20, 21]. Editors and publishers also need to strongly advocate for data sharing to help promote research within their own community. For example, several questions concerning peer review can only be meaningfully answered with access to journal peer review reports. By analogy to clinical data, we think that publicly funded peer review (many researchers are publicly funded) data are a public good, produced in the public interest, and that they should be openly available to the maximum extent possible. While access is possible in some open access journals, all journals need to develop a mechanism to ensure access to all of their peer review reports. It would be unfortunate to advocate for data sharing in clinical medicine and neglect similar needs of journalology researchers.

How might a journal research network move forward? One possibility is to use a template similar to that successfully used by the Committee on Publication Ethics. We have started the network with the development of a website. We hope this will facilitate international outreach, such as to the Counsel of Science Editors, Asia Pacific Association of Medical Journal Editors, and other groups and interested individuals. A few of the network ideas possibly overlap with those of the Responsible Research and Innovation movement [22]. Links to this group and others is also important to pursue.

We hope publishers, editors, funders, and others will join the network. The small group of editors initially approached will speak shortly and will reach out more broadly thereafter. Like any new initiative, we will have to work hard to keep the network practical and of interest to a very broad spectrum of editors and publishers, and funding opportunities will need to be sought to facilitate the initial development of the network and sustain it. Journals participating in research will also need intellectual and fiscal support to participate in research studies.

Journals need best evidence to inform their best practice, which will increase confidence in how journals function as well as help reduce the enormous waste in the system. We invite interested journals and their publishers to join us on this adventure.

## Box 1

1. Does scientific editor training in core competencies result in more use of reporting guidelines by journal editors and peer reviewers?
2. Do reporting guidelines improve the completeness of reporting observational studies?
3. Does a formal assessment of clinical trial protocols during peer review of completed trial reports reduce switched outcomes when these reports are published?
4. Does better peer review reduce the number of retractions?
